# MARCKS and MARCKS-like proteins in development and regeneration

**DOI:** 10.1186/s12929-018-0445-1

**Published:** 2018-05-22

**Authors:** Mohamed El Amri, Una Fitzgerald, Gerhard Schlosser

**Affiliations:** 1 Centre for Research in Medical Devices (CÚRAM), National University of Ireland, Galway, Biomedical Sciences Building, Newcastle Road, Galway, Ireland; 2 Galway Neuroscience Centre, School of Natural Sciences, Biomedical Sciences Building, National University of Ireland, Newcastle Road, Galway, Ireland; 3 School of Natural Sciences and Regenerative Medicine Institute (REMEDI), National University of Ireland, Galway, Biomedical Sciences Building, Newcastle Road, Galway, Ireland

**Keywords:** MARCKS, MARCKS-like protein, Development, Regeneration, Cell migration

## Abstract

**Background:**

The Myristoylated Alanine-Rich C-kinase Substrate (MARCKS) and MARCKS-like protein 1 (MARCKSL1) have a wide range of functions, ranging from roles in embryonic development to adult brain plasticity and the inflammatory response. Recently, both proteins have also been identified as important players in regeneration. Upon phosphorylation by protein kinase C (PKC) or calcium-dependent calmodulin-binding, MARCKS and MARCKSL1 translocate from the membrane into the cytosol, modulating cytoskeletal actin dynamics and vesicular trafficking and activating various signal transduction pathways. As a consequence, the two proteins are involved in the regulation of cell migration, secretion, proliferation and differentiation in many different tissues.

**Main body:**

Throughout vertebrate development, MARCKS and MARCKSL1 are widely expressed in tissues derived from all germ layers, with particularly strong expression in the nervous system. They have been implicated in the regulation of gastrulation, myogenesis, brain development, and other developmental processes. Mice carrying loss of function mutations in either *Marcks* or *Marcksl1* genes die shortly after birth due to multiple deficiencies including detrimental neural tube closure defects. In adult vertebrates, MARCKS and MARCKL1 continue to be important for multiple regenerative processes including peripheral nerve, appendage, and tail regeneration, making them promising targets for regenerative medicine.

**Conclusion:**

This review briefly summarizes the molecular interactions and cellular functions of MARCKS and MARCKSL1 proteins and outlines their vital roles in development and regeneration.

## Background

The Myristoylated Alanine Rich C-Kinase Substrate (MARCKS) is a ubiquitous, highly conserved protein among vertebrates, which is essential for postnatal survival [1], and has been widely studied for its functions in the brain and nervous system. Being highly expressed in nervous tissue, particularly during early development but persisting in the adult, it plays numerous roles related to brain growth, neuronal migration, neurite outgrowth, neurotransmitter release, and synaptic plasticity (reviewed in [2]). In addition, the protein has been implicated in the regulation of other developmental events, including gastrulation [3], myogenesis [4], and vasculogenesis [5].

MARCKS protein has become established as a key regulator of many molecular interactions, such as those involving the dynamic actin cytoskeleton or membrane phosphoinositides (reviewed in [2, 6–8]). Many of the molecular characteristics of MARCKS are also shared by MARCKS-related proteins, including proteins with significant homology in the effector domain such as MARCKS-like protein 1 (MARCKSL1) and other proteins that have similar biochemical functions and localisation patterns, such as growth associated protein 43 (GAP43) and cytoskeletal-associated protein 23 (CAP23) [9]. Whereas GAP43 and CAP23 have long been shown to play important roles in neural regeneration [10, 11], only recently have MARCKS and MARCKSL1 been implicated in regeneration of neural and other tissues [12–14]. This review focuses on the emerging roles of MARCKS and MARCKS-like proteins in development and regeneration and explores possible mechanisms underlying their function.

## Main text

### Domain structures and molecular properties

**Fig. 1:**
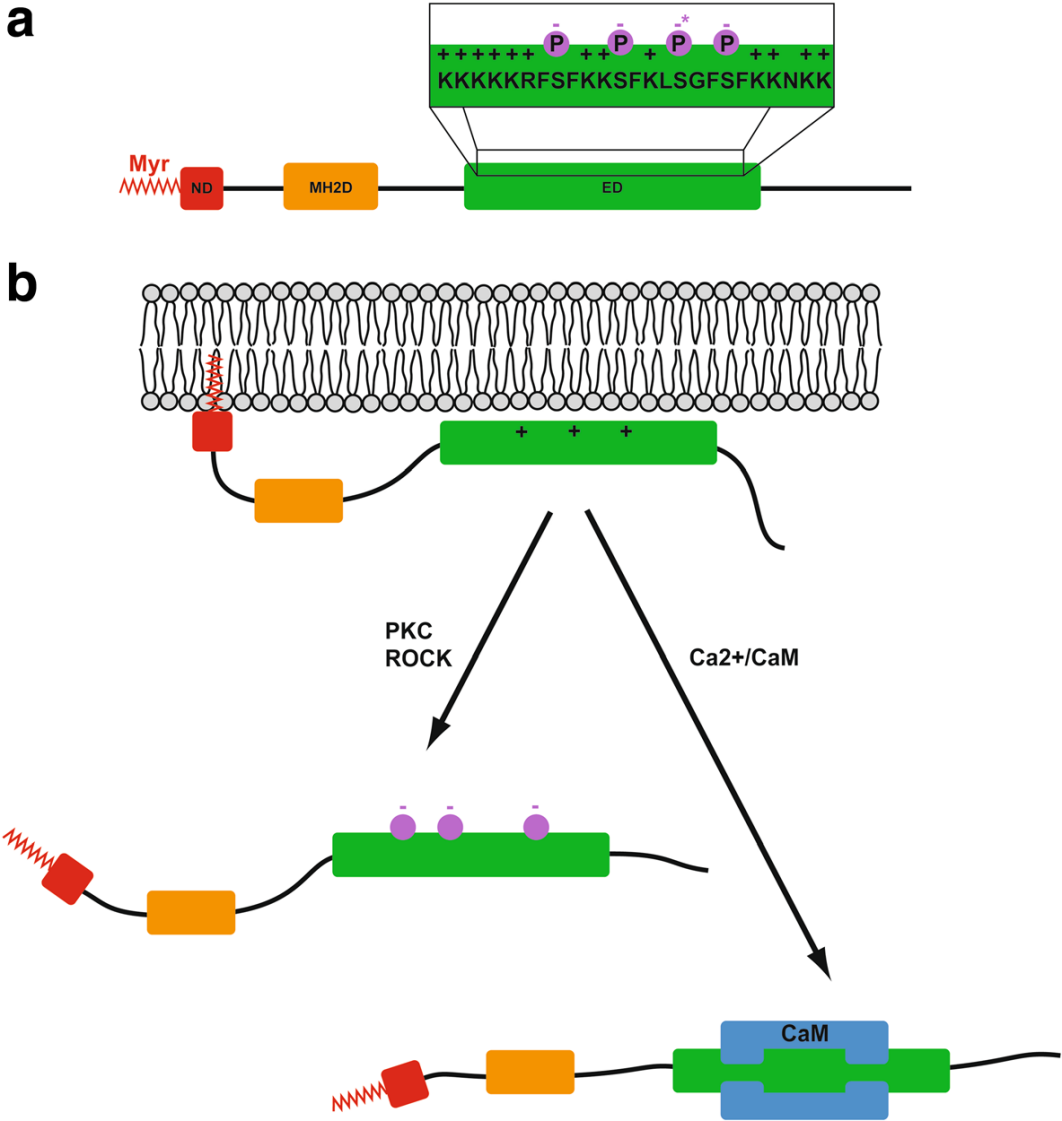
MARCKS protein structure and electrostatic switch. **a** MARCKS protein has three protein domains, the N-Terminal domain (ND), which can be myristoylated (Myr), an MH2 domain (MH2D) and an effector domain (ED). The ED (amino acids 152–176 in human MARCKS) is magnified in the inset showing that it is highly positively charged and has 4 potential phosphorylation sites, one of which (asterisk) is poorly phosphorylated. **b** In the unphosphorylated state and in the absence of Calcium-calmodulin (CaM) binding, MARCKS is tethered to the membrane but becomes released into the cytosol when phosphorylated by protein kinase C (PKC) or Rho kinase (ROCK) or after Calcium-CaM binding. Modified from [8]

MARCKS is an abundant, rod-shaped protein of 35 kDa [15], with three highly conserved functional domains [2, 16] (Fig. 1a). In the centre of the protein, the effector domain (ED) is rich in positively charged lysine residues, while multiple serine residues make it susceptible to phosphorylation by protein kinase C (PKC), or other protein kinases such as Rho kinase (ROCK) [2, 15, 17, 18]. Adjacent to the ED are two highly conserved regions. The first is the MARCKS Homology 2 (MH2) domain [19]. The second conserved region is the N-terminal domain containing a myristoylation site, which undergoes a reversible co-translational attachment of myristic acid to its N-terminal glycine residue [20]. In its non-phosphorylated state, the positively-charged ED attaches to the negatively charged cytosolic face of the plasma membrane [2] (Fig. 1b). As a result, the N-terminal myristoylation site reversibly inserts into the plasma membrane, serving as a lipid anchor for the protein [21, 22]. Once the ED is phosphorylated, it loses its affinity for the plasma membrane, shifting MARCKS back into the cytoplasm [2] (Fig. 1b). This translocation, termed the ‘electrostatic switch’ [22], can also be achieved through increased Ca^2+^ levels, which enable calmodulin to bind to the ED of MARCKS [23] (Fig. 1b).

MARCKS-like protein 1 (MARCKSL1), also known as MARCKS-like protein (MLP), MARCKS-related protein (MRP), Brain Protein F52, or MacMARCKS, shares strong homology and functionality with MARCKS [24]. The 20 kDa protein has a very similar ED to that of MARCKS, which also binds F-actin, Ca^2+^/calmodulin, and acidic phospholipids. In addition, MARCKSL1 contains the same N-terminal myristoylation consensus sequence found in MARCKS [25]. However, it is important to note that MARCKSL1 has a lower alanine content than MARCKS, resulting in potential functional differences, and a distinct distribution pattern in the brain [24].

**Fig. 2:**
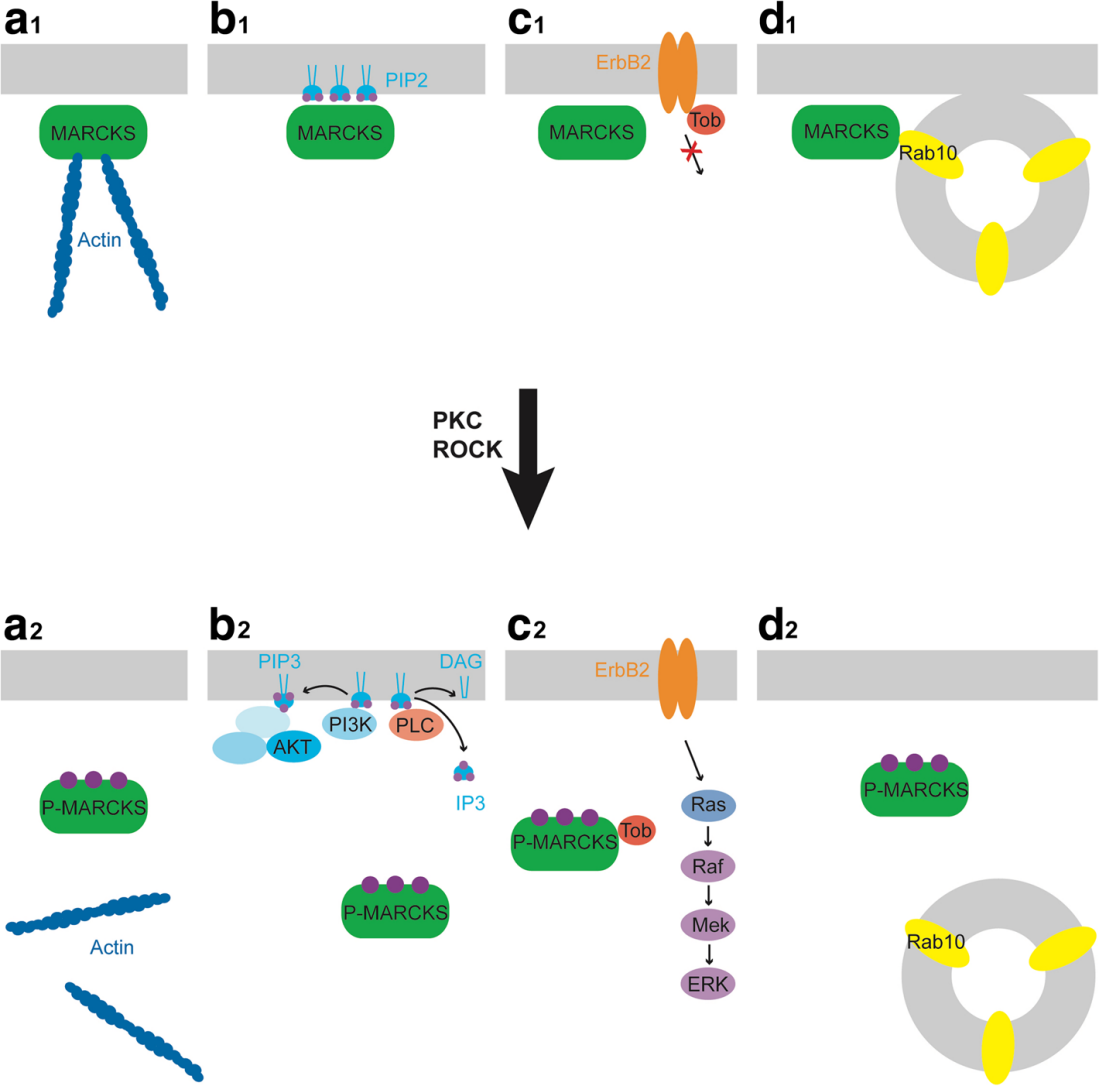
Molecular interactions of MARCKS. Role of membrane- tethered, unphosphorylated MARCKS (**a**_1_-**d**_1_) is compared with its cytosolic, phosphorylated form (**a**_2_-**d**_2_). Membranes are depicted in grey; phosphorylation is indicated by purple circles. **a** Direct actin binding of unphosphorylated MARCKS. **b** PIP_2_ sequestration of unphosphorylated MARCKS; upon phosphorylation of MARCKS, PIP_2_ becomes accessible to PLC and PI3K. **c** Phosphorylated MARCKS binds to Tob resulting in activation of ErbB2 signalling. **d** Unphosphorylated MARCKS binds to Rab10 promoting exocytosis of vesicles. See text for details

Depending on their phosphorylation state, MARCKS or MARCKSL1 have been shown to engage in a number of different molecular interactions. First, when the ED of MARCKS is unphosphorylated and attached to the plasma membrane, it achieves cross-linking of actin filaments by directly binding to filamentous (F) actin [26, 27] (Fig. 2a). In addition, MARCKS can promote the polymerisation of actin [28]. In a similar way, MARCKSL1 bundles and stabilises F-actin upon phosphorylation, increasing filopodium dynamics [29]. These direct interactions with the cytoskeleton have been implicated in the regulation of cell migration in various developmental contexts (see below) as well as in the regulation of mucin secretion in the human bronchial epithelium. The latter process, which is dysregulated in asthma and other respiratory diseases, involves the dephosphorylation of cytoplasmic MARCKS, promoting its interaction with both F-actin and membrane bound proteins of secretory vesicles and resulting in increased mucin secretion [30, 31].

The electrostatic switch mechanism of MARCKS and MARCKSL1 also has important consequences for their ability to interact with phosphatidylinositol 4,5-bisphosphate (PIP_2_) [7, 32, 33]. PIP_2_ is a membrane component, with numerous cellular functions, including second messenger generation and membrane-anchoring of various proteins, including kinases and proteins with MARCKS-like domains [34, 35]. PIP_2_ is either selectively hydrolysed by phospholipase C (PLC), producing inositol triphosphate (IP_3_) and diacylglycerol (DAG) [36], or is further phosphorylated by phosphoinositide 3-kinase (PI3K) to form PIP_3_. These three products act as second messengers in many eukaryotic signal transduction cascades. For example, DAG activates several PKC isozymes, stimulating the phosphorylation of select proteins by PKC. On the other hand, IP_3_ regulates the cytoplasmic concentration of Ca^2+^ by gating a Ca^2+^ channel in the endoplasmic reticulum. Furthermore, IP_3_ functions as a rate-limiting substrate in the synthesis of additional inositol polyphosphates, which can stimulate various protein kinases, transcription, and mRNA processing events [36–38]. PIP_3_, finally, is involved in activating the AKT signalling pathway with a plethora of diverse functions [39].

It has been recurrently shown that membrane-bound MARCKS can isolate and sequester PIP_2_ within specific membrane micro-domains, or “lipid rafts”, for participation in later signal transduction events, suggesting that it can modulate PIP_2_-dependent cellular processes by controlling the spatial availability of the phospholipid for enzymes such as PLC and PI3K [34, 40–42] (Fig. 2b). While PIP_2_ is critical for the activity and localisation of several membrane associated proteins, including focal adhesion kinase (FAK) [34, 35] many of the PIP_2_-dependent processes that MARCKS modulates remain currently unknown. However, PIP_2_ sequestration by MARCKS and related proteins has been shown to promote axon outgrowth [43]. While the mechanism is not completely resolved, it has been proposed that unmasked PIP_2_ interacts with and inhibits proteins promoting actin dynamics (e.g. gelsolin, cofilin, profilin), thereby indirectly stabilizing the cortical actin cytoskeleton. After sequestration of PIP_2_ by MARCKS, these proteins are released and now promote cell motility [43].

A further PIP_2_-dependent process that is affected by MARCKS is the activation of phospholipase D (PLD) [7, 44, 45], which is involved in cytoskeletal actin dynamics, membrane trafficking, cell migration, and mitosis [46–51]. Since PIP_2_ is required for PLD activation, it has been proposed that MARCKS-mediated PLD activation results from the phosphorylation-induced release of PIP_2_ [7]. PLD acts by hydrolysing phosphatidylcholine (PC), producing choline and phosphatidic acid (PA), which serve as second messengers in many signal transduction cascades [52, 53]. For instance, PA is known to play a significant role in actin stress fibre formation [54, 55], vesicular trafficking [56], cell proliferation [57, 58], neurite outgrowth [59], and MAP-kinase activation [60]. In addition, PA can also be converted into DAG and lysophosphatidic acid (LPA) [61], a potent signalling molecule with functions such as neurite retraction [62] and cell proliferation [63].

In addition to its PIP_2_-dependent modulation of various signalling pathways, MARCKS affects other signalling pathways by different mechanisms. As an example, following phosphorylation by PKC, MARCKS activates an ErbB2-mediated signal pathway, by binding to the anti-proliferative negative cell-cycle regulator Transducer of ErbB2 (TOB2), thereby decreasing its affinity to ErbB2 [64] (Fig. 2c). This in turn, promotes cell proliferation and maintenance of normal radial glial identity [65]. In addition, the exogenous overexpression of ErbB2 induces mature astrocytes to become radial glial progenitors in the adult mouse brain, promoting both neurogenesis and targeted neuronal migration [66]. Furthermore, MARCKS has been associated with polysialic acid (PSA), which influences neural differentiation, migration [67] and axonal commissure formation [68–70]. When PSA is added to neural cell adhesion molecules (NCAMs) as a post-translational modification, it co-localises with MARCKS in the plasma membrane, stimulating neurite outgrowth [71]. Moreover, MARCKS has recently been shown to modulate Netrin-1 - Deleted in Colorectal Cancer (DCC) signalling by disrupting the localisation patterns of two of its subcellular mediators, proto-oncogene tyrosine-kinase SRC and FAK. As a result, axonal navigation in the corpus callosum becomes aberrant during a crucial phase of mouse brain development [72].

The apical localisation of MARCKS in ependymal and radial glial cells [73, 74] and the displacement of cell-polarity proteins such as aPKC, PAR3, CDC42, as well as β-catenin, prominin, and N**-**cadherin in *Marcks*^−/−^ mouse embryos [73], suggest that MARCKS is also able to interact with membrane-associated proteins related to cell polarity and anchor them apically, although evidence for direct protein-protein interactions is currently lacking. However, radial glial cell polarity is perturbed in *Marcks*^−/−^ embryos, resulting in reduced proliferation, changes in the proportion of asymmetric cell divisions, and displacement of radial glia cells, which can act both as neural progenitor cells and as pro-migratory scaffolds for neurons in the developing cortex [2, 73]. In addition, MARCKS may also affect cell polarity via PIP_2_-dependent mechanisms [73, 75–77].

Finally, MARCKS has been shown to interact with various vesicular proteins. Direct interactions with Rab10 in plasmalemmal precursor vesicles (PPVs) provide membranes to outgrowing axons when the ED is not phosphorylated [78] (Fig. 2d). Interactions with other vesicle associated proteins such as synapsin [79] or various chaperones [31] have been described and may contribute to the role of MARCKS for secretion of mucin, neurotransmitters, as well as inflammatory cytokines [31, 80–83]. MARCKS probably affects secretion by several distinct mechanisms, since the unphosphorylated form of the protein promotes mucin secretion [30, 31], while the phosphorylated form promotes neurotransmitter release and gut peptide secretion [80, 81].

In summary, MARCKS interacts with numerous molecular pathways. Much less is known about MARCKSL1, but the overall consequences of its interactions appear to be similar to MARCKS. Most notably, MARCKS affects cytoskeletal rearrangements, various signalling pathways, and vesicular trafficking. As a consequence, the protein affects predominantly cellular processes relying on these pathways during development or in the adult such as cell migration, secretion and phagocytosis, and cell proliferation and differentiation. Cell migration is affected not only by MARCKS’ capacity for direct actin binding but also by multiple downstream effects of PIP_2_ sequestration [26, 27, 43, 44]. Secretion and phagocytosis likewise appear to be modulated by several MARCKS interacting factors [31, 78, 79]. Finally, cell proliferation and differentiation are modulated by MARCKS’ interaction with various signalling pathways, as well as possibly its interactions with cell polarity proteins [64, 73, 84].

A large body of evidence indicates that the electrostatic switch mechanism between membrane bound (unphosphorylated) and cytosolic (phosphorylated or CaM-bound) MARCKS plays a crucial role in the regulation of each of these processes. However, there is conflicting evidence for the precise role of membrane bound versus cytosolic MARCKS. As discussed above, secretion appears to be promoted by unphosphorylated, membrane bound MARCKS in some contexts [30, 31] but by phosphorylated, cytosolic MARCKS in other contexts [80, 81]. Similarly, unphosphorylated, membrane bound MARCKS or MARCKSL1 have been shown to promote lamellipodium formation, axon outgrowth and cell motility in neurons and cancer cells in some studies [29, 43, 85–87], whereas phosphorylated, cytosolic MARCKS has been shown to promote cell motility in other studies [88–92]. Moreover, neither phosphorylation of the ED nor myristoylation of MARCKS are necessary for normal gross brain morphology in a transgenic line of mice overexpressing MARCKS [93–95], whereas myristoylation, but not phosphorylation, of MARCKS is required for radial glial polarity and localisation [73].

While some of these apparently paradoxical findings may be due to context-dependent interactions of MARCKS with different binding partners, others may reflect the dynamic requirement of both phosphorylated and unphosphorylated forms of MARCKS. Indeed, phosphorylation of MARCKS in migrating muscle precursors and neutrophils has been shown to be transient, followed by rapid dephosphorylation. While the phosphorylated form permits initial adhesion, the dephosphorylated form of MARCKS supports later stages of cell spreading [96, 97].

### Role of MARCKS and MARCKSL1 in development

MARCKS and MARCKSL1 are expressed almost ubiquitously during vertebrate development, from early developmental stages and onwards, although there are some differences in MARCKS and MARCKSL1 expression patterns between species, developmental stages, tissues, and the phosphorylation state of the proteins. MARCKS and MARCKSL1 mRNA were shown to be maternally supplied in anamniotes and continue to be expressed throughout cleavage and gastrulation [16, 98, 99]. After neurulation, expression of MARCKS and MARCKSL1 is upregulated in the central (CNS) and peripheral nervous system (PNS) of all vertebrates, but continues to be expressed in many mesodermal and endodermal tissues [16, 24, 98–102]. During embryonic development of the CNS, MARCKS is first upregulated in the neuroepithelial cells of the emergent neural tube [100], before localising into the apical membranes of ventricular-zone neural progenitor cells (NPCs) [73, 103]. Subsequently, it is found particularly enriched in axons and dendrites [104, 105].

The nearly ubiquitous expression pattern of MARCKS and MARCKSL1 suggests that they play a vital role during vertebrate development and this is supported by many functional studies. For example, five gene-knockout studies in mice have shown that MARCKS and MARCKSL1 are both required for embryogenesis [1, 24, 100, 106, 107]. According to these reports, the absence of MARCKS and MARCKSL1 interfered with neural tube closure, leading to spina bifida and exencephaly, which resulted in perinatal lethality [108]. In addition, the disruption of the *Marcks* gene led to severe neuromuscular defects and decreased body size in mice [1, 73, 93, 100]. Other neural embryonic defects included agenesis of forebrain commissures (e.g. the corpus callosum), neuronal ectopia, and abnormal retinal/cortical laminations [1, 100].

Additional functional studies in frog and zebrafish have shown that MARCKS plays an important role during early embryonic events such as gastrulation [3]. For example, by blocking MARCKS protein synthesis in *Xenopus* embryos using antisense morpholino oligonucleotides (MO), Ioka et al. reported impaired convergent extension movements due to cytoskeletal deregulation [3]. In zebrafish embryos, blocking the two MARCKS paralogs *marcksa* and *marksb* also resulted in gross phenotypic defects, including severely curved and truncated tails, gill-formation abnormalities, skeletal muscle deformities, and an abnormal brain architecture [16].

The neural abnormalities observed in MARCKS mutants strongly suggest that MARCKS has multiple roles in the developing nervous system. For example, it maintains normal radial glial cell polarity and cell adhesion in the neocortex during brain development [73]. Since mice with mutant non-myristoylatable MARCKS [94] were only partially rescued from severe cranial defects and perinatal death in comparison with mice lacking MARCKS PKC-phosphorylation sites [109], it can be speculated that the function of radial glial cells depends on MARCKS myristoylation rather than phosphorylation [73]. Similarly, in another study, a phosphorylation deficient mutant form of MARCKS protein was able to rescue CNS defects observed in *Marcks*^−/−^ knockout mice, suggesting that phosphorylation of MARCKS is not essential for CNS development [93].

However, another study suggests that the phosphorylation status of MARCKS plays a significant role in spinal cord development. In this study, Garrett et al. conditionally blocked the γ-Protocadherin allele Pcdh-γ, creating high levels of PKC that phosphorylated MARCKS [110]. As a result, dendrite complexity and arborisation were drastically reduced, having severe implications on CNS development. To confirm these results, dendrite abnormalities were rescued by blocking PKC, PLC, and FAK, the latter of which binds to γ-Protocadherins.

Moreover, MARCKS has been implicated in the regulation of neuronal migration and axon outgrowth during PNS and CNS development by modulating growth cone adhesion [85, 111] and the dynamics of the actin cytoskeleton [29, 43]. The latter results in the stimulation of lamellipodia formation and neurite outgrowth by dephosphorylated MARCKS [29, 86, 112].

MARCKS and MARCKSL1 are also implicated in cell adhesion and migration of neural crest cells (NCCs). NCCs are a group of transient migratory cells that originate from the neural tube during embryogenesis and give rise to a variety of different cell types, including sensory neurons and glial cells of the PNS, cranial cartilage, and bone [113, 114]. It has been suggested that migrating-precursor cells of the PNS that originate from NCCs express a significantly higher amount of MARCKS in chick embryos [102]. In addition, mice lacking MARCKSL1 have been shown to have impaired NCC migration, contributing to severe abnormalities including exencephaly and neural-tube defects [107]. For future experiments, it would be interesting to trace the behaviour and fate of MARCKS and MARCKSL1 in NCCs using cell-adhesion and cell-migration assays to further elucidate their role in development and regeneration.

In addition, MARCKS and MARCKSL1 are also involved in modulating migration during development of many other tissues. For example, by reversibly blocking MARCKS-translocation and myoblast-fusion in chick embryos, Kim et al. found that PKC-controlled MARCKS translocation is a prerequisite for myoblast fusion, a key cellular event that shapes the formation, fusion, and repair of embryonic muscle cells [4, 109, 115]. Moreover, MARCKS regulates vascular endothelial cell migration by influencing insulin-dependent signalling to PIP_2_, which in turn affects actin assembly and cellular development in the vascular endothelium [5]. MARCKS has also been shown to play a critical role in angiotensin-II signalling, which directly influences endothelial cell motility [116].

As a whole, the role of MARCKS in cell migration, secretion, proliferation and differentiation appears common to a diverse array of developmental functions, and continues to be important in several adult tissues. For example, as discussed above, adult MARCKS plays an important role in mucin secretion in the airways [30]. Moreover, in the mature nervous system, MARCKS and MARCKSL1 serve a variety of functions, including the promotion of neurotransmitter release and gut peptide secretion [80, 81], as well as a role in synaptic plasticity and learning and memory, possibly due to their ability to promote dendritic spine maintenance [2, 105, 117]. In addition, MARCKS and MARCKSL1 play important roles in the immune system, where they promote migration of inflammatory cells and the secretion of cytokines as discussed in more detail below. Dysregulation of MARCKS or MARCKSL1 has also been implicated in many different cancers, where they affect tumorigenesis, metastasis and angiogenesis [8, 118].

### Role of MARCKS and MARCKSL1 in regeneration

There is strong evidence for a role of the proteins GAP43 and CAP23 in regeneration in both the PNS and CNS [10, 11, 119–123]. These two proteins are structurally and mechanistically related PKC substrates that share numerous functions including PIP_2_- and- actin cytoskeletal regulation with MARCKS [9, 43, 124]. Based on these similarities, the trio of GAP43, MARCKS and CAP23 is commonly referred to as GMC proteins.

For example, GAP43 and CAP23 are highly expressed in mouse motor nerves during regeneration [120, 125] and play a critical role in regulating nerve sprouting [124, 126]. They have also been implicated in the regeneration of axons in the dorsal root ganglia and sciatic nerves [43, 127], olfactory epithelia [128], retinal ganglion cells [129], and the cerebral cortex [130]. Co-expression of these two proteins triggers a 60-fold increase in dorsal root ganglion axon regeneration after spinal cord injury in mice [131]. In the cerebellum, overexpression of GAP43 induces axonal sprouting [132, 133], while downregulation of GAP43 by RNAi interferes with axonal regrowth after injury [134].

In contrast to other GMC proteins, MARCKS and MARCKSL1 have only recently emerged as potentially important players during the regenerative process. In 2000, McNamara et al. showed that MARCKS expression, like GAP43, is significantly upregulated in regenerating neurons after facial axonal lesions in rats [12]. Both proteins are also highly expressed during neurite outgrowth of dorsal root ganglia and superior cervical ganglia [123]. Furthermore, MARCKS is highly upregulated during optic nerve regeneration [135] and during axonal sprouting after brain stroke [126], suggesting that MARCKS, like GAP43 and CAP23, may play an important role in axon outgrowth during regeneration in both PNS and CNS, although disappointingly, functional studies confirming this are still lacking. Outside of the nervous system, MARCKS has also been shown to be upregulated during lens regeneration [136] and during cardiac tissue regeneration following infarction [137], while MARCKSL1 is elevated during lungfish fin regeneration [14]. Using qPCR analysis on the fin blastema, a collection of relatively undifferentiated and proliferating cells capable of regeneration, it was shown that lungfish *Marcksl1* reaches its highest level of expression 1 day post amputation, returning to basal levels at 3 weeks post-amputation [14].

A recent publication by Sugiura et al. now suggests a different and more pervasive role for MARCKSL1 in regeneration, by demonstrating that extracellularly released axolotl MARCKS-like protein (AxMLP) is responsible for inducing the early proliferative response in axolotl tail and limb regeneration [13]. Using a variety of experimental strategies, these researchers identified AxMLP as an extracellular factor that is strongly associated with cell proliferation and blastemal length. For instance, in-vivo knock-down studies revealed that AxMLP is necessary for the elevated levels of cell proliferation following injury, while immunohistochemical analysis of AxMLP distribution in epidermal and spinal cord tissues showed that the protein is mostly cytoplasmic in uninjured tissue, before translocating to the membrane following injury in accordance with its proposed extracellular secretion.

Today, the mechanisms of AxMLP extracellular release remain currently unknown. Confirmation and elucidation of the mode of MARCKSL1 secretion promises to provide novel insights into unconventional protein secretion mechanisms since it does not contain a signal peptide. In addition, secretion of MARCKS or MARCKSL1 so far has only been reported in the highly regenerative axolotl but has not been found in other vertebrates, raising the possibility that secretion of these proteins may be linked to their ability to promote regeneration.

Taken together with previous observations, this study suggests that MARCKS and MARCKSL1 play important roles during regeneration. While the underlying molecular mechanisms are still unresolved, insights from molecular and developmental studies suggest some candidate pathways, which will be discussed in the following sections. As summarized above, MARCKS and MARCKSL1 have important roles in the development of multiple tissues, but they have also been shown to be important in the inflammatory response to injury. This suggests that MARCKS and MARCKSL1 may potentially affect the regenerative response in two very different but not mutually exclusive ways. First, by promoting the regenerative process in the injured tissue itself and second, by modulating the inflammatory response.

#### Potential direct roles in the injured tissue

The first response to limb- or tail amputation in species capable of regeneration is the formation of a blastema, involving the migration of cells, followed by cell-cycle re-entry and blastemal-cell proliferation. These cellular mechanisms, including migration, proliferation, and differentiation, are known to be conserved amongst most species after injury [138]. MARCKS and MARCKSL1 have been implicated in many of the processes underlying blastema formation, suggesting that they may play multiple roles during this process. First, MARCKS has been shown to promote proliferation, by activating the ErbB2-mediated signal pathway or by interacting with cell polarity proteins [64, 73]. As mentioned previously, ErbB2 overexpression has also been shown to induce astrocytes to dedifferentiate and revert to a progenitor state [66] and similar processes would be required during blastema formation.

Second, MARCKS has been shown to induce cellular migration, which is another critical component of regeneration [139–141]. As discussed above, MARCKS influences cell migration by a multitude of mechanisms, including its interactions with actin, PIP_2_ sequestration, and the activation of various signalling pathways. In addition, MARCKS has been shown to mediate the effects of the noncanonical Wnt pathway on cortical actin dynamics during the formation of lamellipodia- and filopodia-like protrusions [3]. This pathway has been shown to promote regeneration in *Xenopus* and zebrafish, and is necessary for axolotl appendage regeneration [142]. The established role of MARCKS in promoting axon outgrowth via its effect on cell adhesion and actin dynamics may also contribute to neural regeneration.

#### Potential indirect roles in modulating inflammation

The process of inflammation also plays a critical role in the regeneration of injured tissue through a variety of highly conserved pathways. Although severe inflammation typically inhibits regeneration, a moderate and well-regulated inflammatory response may actually be required for the initiation of regeneration [143–145]. Depending on the injury site and organism, cells such as macrophages and neutrophils that infiltrate the wound and secrete cytokines are characteristic of the inflammatory response [139, 144, 146–150]. Macrophages, which are necessary for salamander limb regeneration [151, 152], are known to functionally depend on MARCKS and MARCKSL1.

The importance of MARCKS and MARCKSL1 in inflammation is well established. MARCKSL1 was initially termed ‘MacMARCKS’ due to its high level of expression in macrophages [153], and up to date, numerous studies have associated MARCKS and MARCKSL1 with normal macrophage function [154, 155]. For instance, both MARCKS and MARCKSL1 are implicated in macrophage transmigration [156] through a process involving phosphorylation, actin binding, and cytosol translocation [90]. During the inflammatory response, MARCKS has also been shown to act as a major regulator of human neutrophil migration and adhesion [88], also promoting neutrophil secretion of inflammatory cytokines [82, 83, 157, 158].

Finally, MARCKS upregulation is associated with microglial activation and neuroinflammation after CNS injuries [159]. Interestingly, although the mechanisms of microglial activation during axonal regeneration remain disputable [160, 161], studies suggest that amyloid beta might be responsible for promoting microglial activation by stimulating PKC and MAPK to phosphorylate MARCKS [162].

## Conclusions

Over the past three decades, major advances in research have identified MARCKS and MARCKSL1 as key players during developmental and regenerative processes. These include brain, kidney, blood-vessel, and muscle development, as well as appendage regeneration. However, while numerous molecular interactions of MARCKS-related molecules, such as their interactions with the actin cytoskeleton and membrane phosphoinositides have been unravelled, their respective role for various developmental and regenerative processes is very poorly understood. Moreover, these multifaceted molecules probably contribute to development and regeneration by additional mechanisms which remain yet to be characterised.

For example, the phosphorylation-site domain (ED) in MARCKS is homologous to a region in diacylglycerol kinase zeta (DGKζ), which has been shown to bind to and modulate the function of retinoblastoma protein (Rb) [163–165]. Rb is implicated in cell cycle regulation and has been shown to be important for cell cycle re-entry of newt myotubes [166–168], but whether this function is MARCKS or MARCKSL1 dependent has not been investigated yet. The ED also acts as a nuclear localisation signal, suggesting that MARCKS may have unrecognized functions in the nucleus, including potential roles in the modulation of gene expression and of nuclear PIP_2_ localisation [169].

Retinoic acid (RA) is another factor which has been shown to affect MARCKS function in rat hippocampal cells, where it leads to its translocation from the membrane to the cytosol [170]. As a metabolite of vitamin A, RA plays a significant role in numerous regenerative processes such as nerve, auditory hair cell, fin/limb and lung regeneration [171–177], but whether any of these effects depend on MARCKS remains yet to be explored. Finally, the demonstration that AxMLP acts as a secreted factor in axolotl limb regeneration suggests additional and hitherto unknown modes of action.

Therefore, further research is required to assess the precise mechanisms by which MARCKS and MARCKSL1 contribute to development and regeneration, providing professionals with the molecular tools that will help them design new therapies for illnesses such as asthma, cancer, and spinal cord injury [8, 131, 178].

## Abbreviations

aPKC: Atypical protein kinase C
AxMLP: Axolotl MLP
CAP23: Cytoskeletal associated protein 23
DAG: Diacylglycerol
DGKζ: Diacylglycerol kinase zeta
ED: Effector domain
FAK: Focal adhesion kinase
GAP43: Growth associated protein 43
hpa: Hours post-amputation
IP3: Inositol triphosphate
LPA: Lysophosphatidic acid
MARCKS: Myristoylated alanine-rich C-kinase substrate
MARCKSL1: MARCKS-like 1
MH2: MARCKS homology 2
MLP: MARCKS-like protein
MO: Morpholino oligonucleotides
MRP: MARCKS-related protein
NCAM: Neural cell adhesion molecule
NCCs: Neural crest cells
NPCs: Neural progenitor cells
PA: Phosphatidic acid
PAR3: Partitioning defective 3
PC: Phosphatidylcholine
PI3K: Phosphoinositide 3-kinase
PIP_2_: Phosphatidylinositol 4,5-bisphosphate
PKC: Protein kinase C
PLC: Phospholipase C
PLD: Phospholipase D
PPVs: Plasmalemmal precursor vesicles
PSA: Polysialic acid
RA: Retinoic acid
Rb: Retinoblastoma protein
ROCK: Rho kinase
TOB2: Transducer of ErbB2
